# Effect of Different Thawing Methods on the Quality and Antioxidant Activity of Frozen *Actinidia arguta*

**DOI:** 10.3390/foods15111920

**Published:** 2026-05-29

**Authors:** Lina Chen, Meijia Li, Mingzhe Yang, Xiaohui Sheng, Tienan Wang

**Affiliations:** Department of Food Science and Engineering, College of Food Science and Engineering, Changchun University, No. 6543, Satellite Road, Changchun 130022, China

**Keywords:** thawing methods, *Actinidia arguta*, water distribution, textural property

## Abstract

This study investigated the effects of air thawing (AT), running water thawing (RWT), microwave thawing (MT), ultrasonic thawing (UT), and high-pressure processing thawing (HPPT) on the physicochemical properties, nutritional content, sensory quality, microstructure, and antioxidant capacity of frozen *Actinidia arguta*. Except for air thawing, the other four thawing methods significantly improved thawing efficiency. MT caused uneven heating and was prone to local overheating. HPPT enhanced color appearance and resulted in higher levels of chlorophyll, β-carotene, flavonoids, ascorbic acid, and polyphenols. However, HPPT reduced water-holding capacity and resulted in a softer texture. Moisture distribution, texture analysis, and scanning electron microscopy revealed that UT achieved uniform thawing without causing severe damage to fruit tissue structure. Consequently, UT-treated *A. arguta* exhibited firmer tissue structure, better water retention, and improved consumer palatability. Regarding antioxidant activity, both UT and HPPT demonstrated significant advantages. In summary, HPPT yields nutritionally enriched *Actinidia arguta*, while UT produces *Actinidia arguta* with superior textural quality. This study provides alternative strategies to conventional thawing methods for frozen *Actinidia arguta*.

## 1. Introduction

*Actinidia arguta* (*A. arguta*), also known as hardy kiwifruit or miniature kiwifruit, is a perennial fruit tree belonging to the kiwifruit family, genus Actinidia [1]. *Actinidia arguta* has a strong potential to improve digestion, anti-inflammatory and antioxidant, prevent cancer, and lower blood pressure due to its richness in biologically active components [2,3,4].

*A. arguta* softens quickly after ripening due to loss of galactose and solubilization of pectin [5]. The soluble solids content of *A. arguta* at the time of commercial harvest typically ranges from about 10% to 14%, and its shelf life at 20 °C lasts only 1–2 weeks if no storage interventions are applied [6]. Small berries, including *Actinidia arguta*, are widely cultivated in China and ripen in concentrated periods. Their high perishability and short shelf life cause serious postharvest waste, highlighting the necessity of effective preservation and deep processing. Compared with drying, frozen storage better maintains fruit quality and bioactive nutrients, and stably supplies high-quality raw materials for industrial production of juice, jam, puree, and functional foods. The additional freezing cost is offset by reduced rot loss and higher product added value. Moreover, this strategy can be extended to other small berries, offering practical references for their industrial storage and comprehensive utilization. Freezing is a traditional and effective means of food preservation and is important for maintaining the quality of seasonal and perishable fruits [7]. Research has found that *A. arguta* retains its original quality for up to six weeks when stored frozen [8]. To maximize the quality of frozen food, it is essential to choose the appropriate thawing method. While traditional methods such as air thawing and water thawing offer the advantage of ease of operation, they also come with obvious disadvantages. These disadvantages include slow thawing speed, microbial proliferation, and irreversible damage to food quality [9]. Common thermal defrosting methods include microwave defrosting and ultrasonic defrosting. These defrosting methods mainly rely on the conversion of energy into thermal energy through the transfer of thermal energy to achieve the purpose of defrosting [10]. Families and restaurants frequently use microwave thawing due to its quick and energy-saving benefits, but the heat conversion is uneven and incompatible with metal packages [11]. Some say it turns acoustic energy into thermal energy, while others say ultrasonic-induced microvapors can improve heat and mass transfer, making the ice/liquid interface less resistant to these processes [12,13]. As a non-thermal defrosting method, high-pressure thawing is not widely used due to the complexity of the operation and the cost of the equipment [14]. High-pressure thawing primarily speeds up the thawing process by decreasing the melting point and specific heat capacity of ice, while simultaneously enhancing its thermal conductivity [15]. The thawing process may affect the water content and color of the fruit. In addition, water loss often leads to the depletion of water-soluble nutrients such as vitamins and carbohydrates [16]. Liu et al. [17] investigated the effects of ultrasonic thawing on mango pulp quality, reporting that while the process significantly accelerated the thawing rate, the outcomes were intensity-dependent. Higher intensities increased phenolic acid content but did not affect carotenoids and ultimately led to a deterioration in sensory quality. Eshtiaghi et al. [18] found that strawberries thawed at a pressure of 600 MPa for 15 min had a higher sucrose content than those thawed for 20 min at atmospheric pressure.

Different thawing methods differ greatly in heat–mass transfer mechanism, temperature rising rate, and processing duration, thereby leading to divergent quality performance of frozen fruit. Traditional air thawing and running water thawing usually require longer processing time, which accelerates tissue respiration, microbial growth, and cell structure damage, ultimately causing severe drip loss and gradual degradation of soluble nutrients and bioactive components. By contrast, microwave and ultrasonic thawing shorten thawing time via energy conversion but may induce uneven thermal distribution and local tissue stress, which inevitably affect texture properties and antioxidant retention. High-pressure thawing alters the phase transition temperature of water and accelerates ice melting under a controlled low-temperature environment, which effectively reduces cell rupture, inhibits nutrient leaching, and better preserves active ingredients.

Nevertheless, existing studies mainly focus on a single thawing technology or conventional berry fruits. For *Actinidia arguta*, there is still a lack of systematic comparative investigation on how typical thawing approaches affect thawing duration, temperature variation, water loss, texture deterioration, and antioxidant compound degradation. The relevant mechanism linking thawing method, microstructure, and nutrient retention remains unclear. This research gap limits the optimization of postharvest preservation and industrial thawing processing of *A. arguta*. Therefore, a comprehensive comparison of five representative thawing methods is necessary to clarify their effects on physicochemical quality, nutritional components, and microstructural characteristics.

Previous studies have shown that the freeze–thaw cycle process will affect the moisture status and physicochemical properties of *A. arguta* [19]. Air thawing (AT) and running water thawing (RWT) are common thawing methods, but they require a long time, result in severe moisture evaporation, and nutrient loss. Although microwave thawing (MT) and ultrasonic thawing (UT) are widely used in rapid thawing applications in daily life, MT easily causes local overheating of food, while UT requires high energy. High-pressure processing thawing (HPPT), as an emerging technology, has received widespread attention, especially for its high nutrient retention and efficiency. In this study, the effects of five thawing methods, namely air thawing (AT, 20 ± 2 °C), running water thawing (RWT, 60 mL/s water flow), microwave thawing (MT, 900 W microwave with intermittent temperature monitoring), ultrasound thawing (UT, 200 W, 40 kHz), and high hydrostatic pressure pulse thawing (HPPT, 100 MPa, 25 ± 2 °C with 10 s rapid pressure release), on the physicochemical properties, nutritional components, sensory quality, and microstructure of frozen *A. arguta* were investigated. The purpose of this study was to explore new high-quality processing methods for such fruits.

## 2. Materials and Methods

### 2.1. Raw Material Handling

*A. arguta* was provided by the College of Horticulture, Changchun University. The *Actinidia arguta* fruits were harvested in September 2024 from the experimental orchard of Changchun University (Changchun, Jilin Province, China) at commercial maturity. Within 1 h after harvest, the fruits were transported to the laboratory under ambient conditions and immediately sorted. Fruits with consistent color, spotless surface, and uniform maturity were selected and washed with distilled water, then the surface was gently dried with dust-free paper. Treated *A. arguta* (size 3.75 ± 0.21 cm, diameter 2.56 ± 0.24 cm) were placed in ziplock bags of 10 each. Subsequently, they were placed in an explosion-proof cryopreservation box (DW-60W138, Haier Biomedical Co., Ltd., Qingdao, China) in the laboratory room at −80 °C for the freezing process, and the center temperature of the samples was detected by a wireless thermometer (EM500-PT100 PT100, Star Controls IOT, Xiamen, China). Upon reaching a core temperature of −20 °C, the samples were transferred to a −20 °C freezer and maintained there for 7 days, after which they were thawed for subsequent measurements.

### 2.2. Thawing Treatment

When thawing, it is ensured that the mass of *A. arguta* treated by each method is similar and the number of single fruits treated is equal. Specifically, approximately 500 g of frozen fruits (50 fruits per batch) were used for each thawing method per repetition. CK: unfrozen *A. arguta*. AT: The samples were thawed at a room temperature of 20 ± 2 °C. RWT: The samples were thawed at a flow rate of 60 mL/s while ensuring that the water flowed evenly over the surface of the fruit. MT: The samples were placed on a flat plate and thawed in a microwave oven (BG720KG4-NA, Midea Kitchen Appliance Manufacturing Co., Ltd., Foshan, China). To avoid overheating of the samples, a fast microwave heating program was used, which was performed at a high power of 900 W, and the center temperature of the samples was observed at intervals of 10 s. The microwave oven operated at a frequency of 2450 MHz and had an input power of 1280 W. UT: Samples were thawed in water using an ultrasonic cleaner (HN-1000Y, Connaught Instruments, Shanghai, China) with a power of 200 W and a frequency of 40 KHz [17]. HHPT: The samples were placed in polyethylene bags and thawed at 100 MPa using a laboratory-specific high hydrostatic pressure apparatus (SHPP-5L, Lite-On Technology Co., Ltd., Taiyuan, China). A rapid pressure relief of 10 ± 1 s was used, and the temperature was 25 ± 2 °C [20]. During the thawing process, the center temperature of the samples was measured using a wireless thermometer, and the thawing time was determined by measuring the time it took for the center temperature of the samples to increase from −20 °C to 0 °C. The thawing time was measured using a wireless thermometer. All tests were completed within 4 h of thawing, and each test was performed in triplicate.

### 2.3. Physicochemical Properties

#### 2.3.1. Determination of pH, Total Soluble Solids (TSS) Content, and Titratable Acid Content (TAC)

The thawed *A. arguta* sap was obtained by squeezing the sap using 2 layers of 100 mesh filter cloth. The pH and TSS contents of the samples were determined using a pH meter (PHS-3C, Yidian Scientific Instruments Co., Ltd., Shanghai, China) and an Abbey refractometer (WYA-2WAJ, Yi-Wei Instruments Co., Ltd., Shanghai, China), respectively. Titratable acid was determined according to Wang et al. [21].

#### 2.3.2. Determination of Drip Loss

The surface of thawed *A. arguta* samples was cleaned well with dust-free paper to properly remove exuded moisture. The weight of the sample before thawing (M_1_) and the weight of the sample after thawing (M_2_) were recorded using an electronic balance (JA2003, Sunyu Hengping Scientific Instruments, Shanghai, China). The value of drip loss was calculated according to the following formula:

$$Drip\;loss\left(\%\right)=\frac{M_{1}-M_{2}}{M_{1}}\times 100\%$$

#### 2.3.3. Water Distribution

Proton relaxation data for fresh and frozen *A. arguta* samples were acquired at 25 °C using a low-field nuclear magnetic resonance (NMR) analyzer (NM42-040H-I, Suzhou Nuomag Electric Co., Ltd., Suzhou, China), following the method of Li et al. [22] with partial modifications. After wiping surface moisture with paper towels, T2 transverse relaxation data were acquired using a Carl-Percival-Meebom-Gill (CPMG) sequence(NM42-040H-I, Suzhou Nuomag Electric Co., Ltd., Suzhou, China) with 90° and 180° pulse durations of 12.52 and 24.00 microseconds, respectively. Additional parameters were set as follows: TW (wait time) 1000 ms, TE (echo time) 0.12 ms, NECH (number of echoes) 18,000, NS (number of scans) adjusted to 4. The raw CPMG decay curves were fitted with a multi-exponential model using the Synchronous Iterative Reconstruction Technique (SIRT) algorithm in MultiExp Inv analysis software(ver4.0) (Niumag Electric Co., Shanghai, China). From this, water distribution and mobility were characterized by integrating the transverse relaxation times (T21, T22, T23) with their corresponding peak area ratios for bound, fixed, and free water (P21, P22, P23).

### 2.4. Nutrients

#### 2.4.1. Determination of β-Carotene Content

The β-carotene content of the samples was determined according to the method of Gloria et al., with minor modifications [23]. A homogenate of 5 g of *A. arguta* was taken and added to 10 mL of petroleum ether-acetone (3:7, *v*/*v*) mixture for extraction, and the extract was refluxed in a water bath three times for 1 h each time. After filtration, the filtrate was concentrated, and the solution was fixed with petroleum ether into a 50 mL brown volumetric flask to be stored in a low-temperature, light-proof bottle. The standard curve was plotted with β-carotene standards, and the absorbance value at 465 nm was determined by using an enzyme labeling instrument (HBS-1096A, DeTie Biotechnology Co., Ltd., Nanjing, China).

#### 2.4.2. Determination of Flavonoid Content

Flavonoid content was determined by the aluminum chloride colorimetric method and calculated using a rutin standard curve [24]. To 5 g of *A. arguta* homogenate, 4 mL of distilled water was added, followed by 5% sodium nitrite solution and 10% aluminum chloride solution in that order. The mixture was incubated for 5 min at room temperature, and then 2 mL of 1 M sodium hydroxide was added to the mixture. Mix well and measure the absorbance value at 510 nm using an enzyme marker. Flavonoid content was calculated using a rutin standard curve.

#### 2.4.3. Determination of Chlorophyll Content

Chlorophyll content was determined as reported by El-Nakhel et al. [25]. Briefly, *A. arguta* homogenates were centrifuged at 12,000× g for 15 min to extract the supernatant for assay. The absorbance of chlorophyll a and b was determined using a UV-visible spectrophotometer (UV-2700, Shimadzu, Kyoto, Japan) at two wavelengths, 663 and 645 nm, respectively. The total chlorophyll was then calculated as the sum of chlorophyll a and b. The total chlorophyll was calculated as the sum of chlorophyll a and b.

chlorophyll a: C_a_ = 12.72 A_663_ − 2.95 A_645_

chlorophyll b: C_b_ = 22.88 A_645_ − 4.67 A_663_

total chlorophyll: C_i_ = C_a_ + C_b_

#### 2.4.4. Determination of Ascorbic Acid Content (AAC)

Ascorbic acid content was determined using an ascorbic acid content determination kit [26] (Qiyi Biotechnology Co., Ltd., Shanghai, China). Briefly, 5 g of the sample homogenate was taken for the assay. The principle was that ascorbic acid could reduce Fe^3+^ to Fe^2+^; o-diazophene and Fe^2+^ would form a red complex with strong absorption at 534 nm, and the absorbance value was directly proportional to the ascorbic acid content in the reaction solution.

#### 2.4.5. Determination of Total Phenolic Content (TPC)

Referring to the method of Dong et al. [27], the total phenolic content was measured by the Folin–Ciocalteu method with slight modifications. Take 5 g of sample homogenate and 5% (*v*/*v*) ethanol-water solution to 100 mL; aspirate 1 mL of the liquid from the homogenate and add 1 mL of Folinol and 4 mL of 7.5% Na_2_CO_3_ solution sequentially. After mixing, the solution was fixed with 60% ethanol in 25 mL of water. The reaction was kept away from light for 1.5 h, and the absorbance value was measured at 765 nm. The polyphenol content was calculated using a gallic acid standard curve.

### 2.5. Sensory Qualities

#### 2.5.1. Determination of Color

The equatorial parts of *A. arguta* were selected for color determination, and all color parameters were averaged over 35 measurements. The CIElab parameters of *A. arguta* were determined using a benchtop spectrophotometer (Shenzhen Sann Technology Co., Ltd., Shenzhen, China), and the instrument should be calibrated for black and white before use. The parameters obtained directly from the instrument were L* (luminance), a* (green/red), and b* (blue/yellow). Hue angle (h°) was calculated as arctan(b*/a*) with quadrant adjustment, and chroma (C) was calculated as √(a^2^ + b^2^). One-way analysis of variance (ANOVA) followed by Tukey’s HSD post hoc test was used to compare h° and C among the six treatment groups. Significance was set at *p* < 0.05. Results are presented as mean ± standard deviation (SD).

#### 2.5.2. Determination of Texture

The method of Chong et al. [28] was referenced to be determined using a CTX000000 texture analyzer (Ametek-Böhlerfeld, Middleborough, MA, USA) with slight modifications. The hardness, adhesiveness, cohesiveness, springiness, and chewiness of *A. arguta* can be derived directly from the instrument.

### 2.6. Scanning Electron Microscope (SEM) Observation

The microstructure of thawed *A. arguta* pulp was observed using a JSM-6510LA scanning electron microscope (JSM-6510LA, JEOL, Tokyo, Japan) with an accelerating voltage of 3 kV at a magnification of 100× [29].

### 2.7. Antioxidant Capacity

#### 2.7.1. DPPH Free Radical Scavenging Capacity

Juice samples, mixed with methanol at a 1:1 (*v*/*v*) ratio, were centrifuged at 12,000× g for 5 min using a benchtop centrifuge (3K15, Sigma, St. Louis, MO, USA). The resulting supernatant was then collected for antioxidant capacity analysis. Following the method of Zhang et al. [30]. DPPH radical scavenging activity was assessed, with results expressed as trolox equivalents (mmol/L).

#### 2.7.2. ABTS Free Radical Scavenging Capacity

The ABTS radical scavenging capacity of the juice samples was determined with reference to the method of Monsibaez et al. [31]. Briefly, the absorbance of the prepared ABTS masterbatch at 734 nm was adjusted to 0.700 ± 0.050 before use to obtain the ABTS working solution. A total of 100 μL of juice-methanol supernatant was mixed with 2 mL of ABTS working solution and incubated at room temperature in a dark environment for 6 min, and the absorbance of the mixture at 734 nm was measured by an enzyme meter. The results were expressed in trolox equivalents mmol/L.

#### 2.7.3. Iron-Reducing Antioxidant Capacity

The FRAP of the juice samples was determined with reference to the method of Pulido et al. [32]. Briefly, 30 μL of sample was mixed with 90 μL of distilled water and 900 μL of FRAP reagent [2.5 mL of 20 mmol/L TPTZ solution] and incubated at 37 °C for 30 min. The absorbance of the mixture was measured at 593 nm using an enzyme meter. The ferrous ions reduced by the juice samples were calculated from the ferrous sulfate calibration curve. The results were expressed in trolox equivalents mmol/L.

### 2.8. Statistical Analysis

The results for texture were averaged over 10 parallels, and all other results were averaged over three parallels. Results are expressed as mean ± standard deviation (SD). Statistical analysis was performed using SPSS 22 (IBM Corporation, Armonk, NY, USA), where significant differences between mean values were determined by the Duncan test at a significance level of *p* < 0.05. Graphical representations were generated with Origin 2022 (Origin Lab Corp., Northampton, MA, USA).

## 3. Results and Analysis

### 3.1. Freeze–Thaw Curve

**Figure 1:**
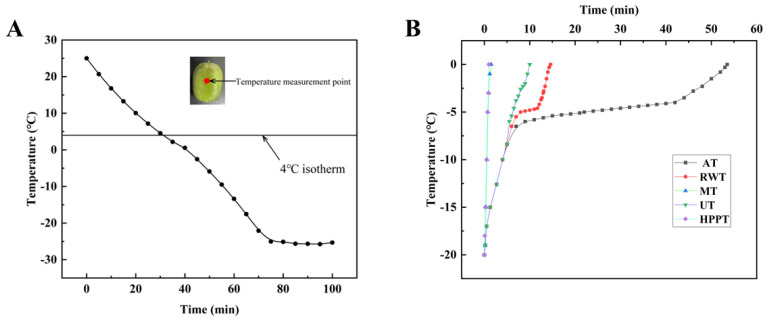
(**A**): Freezing curve of *A. arguta*. (**B**): Thawing profiles of frozen *A. arguta* under various thawing treatments (*n* = 3, with 6 fruits per replicate). (Abbreviations: AT, air thawing; RWT, running water thawing; MT, microwave thawing; UT, ultrasonic thawing; HPPT, high-pressure processing thawing).

The freezing profile of *Actinidia arguta* is presented in Figure 1A. The fruit center temperature decreased from 25 °C to −20 °C within approximately 75 min, exhibiting a two-stage cooling behavior: a gradual decline above 0 °C, followed by a rapid temperature drop once ice nucleation occurred below 0 °C. A slow freezing rate at temperatures just above 0 °C promotes the growth of large extracellular ice crystals, which can mechanically damage cell membranes and exacerbate drip loss during subsequent thawing. By contrast, rapid freezing favors small, evenly distributed ice crystals, better preserving tissue integrity and texture quality. Thawing behavior under different treatments is compared in Figure 1B. A clear hierarchy in thawing efficiency was observed, with HPPT > MT > UT > RWT > AT in terms of thawing rate (*p* < 0.05). Specifically, the time required to reach 0 °C at the geometric center was significantly shorter for HPPT (1.0 min) and MT (1.5 min) than for UT (10.0 min), RWT (14.6 min), and AT (53.5 min). Notably, all treatments exhibited a slow thawing phase within the −5 to 0 °C range, corresponding to the zone of maximum ice crystal formation. The superior performance of HPPT can be attributed to a pressure-induced reduction in the phase-transition temperature of water, which increases the temperature gradient and accelerates heat transfer [15,33]. MT takes slightly longer thawing time than HPPT, but significantly (*p* < 0.05) shorter than the other three defrosting methods. MT defrosts primarily through the conversion of electromagnetic energy to heat [34]. However, the experiment found that the microwave treatment’s effect was closely related to time and sample placement. According to the current research reports, the thawing mechanism of ultrasound has not been clearly determined. However, one claim is recognized: the acoustic energy of ultrasound is converted into thermal energy, thus accelerating the thawing effect of frozen samples. Water has a higher heat transfer efficiency than air [35]. Thus, RWT takes significantly less time than AT. Importantly, thawing speed is closely linked to post-thaw quality: slow thawing (e.g., AT) increases drip loss, accelerates texture softening, and promotes the loss of water-soluble antioxidants, while fast thawing (HPPT, MT) can minimize these adverse effects, thereby better maintaining color, texture, and antioxidant activity.

### 3.2. Physicochemical Properties

#### 3.2.1. pH, TSS, and TAC

Cell structure damage induced by different thawing rates inevitably causes leakage of intracellular electrolytes and organic acids, thereby changing tissue pH. A stable pH environment can effectively alleviate cell membrane oxidation, reduce browning and juice loss, and maintain sensory and nutritional quality. In contrast, abnormal pH fluctuations exacerbated tissue softening, color deterioration, and nutrient loss of *Actinidia arguta*, which ultimately affected the overall post-thaw quality of *Actinidia arguta*. As shown in Table 1, there was no significant difference (*p* > 0.05) in the pH of *A. arguta* from the five thawing methods. pH is a key indicator of fruit acidity and microbial stability, directly influencing sensory quality and shelf life. No significant differences in pH were observed among all thawing treatments (3.5–3.7, *p* > 0.05), indicating that cell membrane damage and the associated release of intracellular ions during thawing were not severe enough to alter the overall acidity of the fruit. TSS is primarily composed of sugars and soluble organic compounds, and it is closely linked to fruit sweetness and consumer acceptance. By contrast, the slower thawing processes of AT and RWT are consistent with previous studies showing that rapid thawing methods enhance the extractability of soluble solids in berries. TSS and TA are two important attributes related to product texture, which affect the taste, flavor, and shelf life of the food products. TSS consists mainly of reducing and non-reducing sugars in either free or bound form, and TA refers to acids such as organic acids and phenolic acids [36]. Soluble sugar constitutes the main component of soluble solids. A higher soluble solid content corresponds to greater sweetness of the fruit. Moderate organic acids combined with high soluble solids endow the fruit juice with a pleasant sweet–sour balance rather than cloying sweetness, which improves consumer acceptability. The TSS of *A. arguta* of MT, UT, and HPPT were significantly higher than those of CK, AT, and RWT (*p* < 0.05). This is mainly due to the higher heating rate of microwave, the cavitation effect of ultrasound, and the destruction of cell walls and cell membranes by ultrahigh pressure to promote the rapid release of intracellular soluble solids. In addition, the tissue structure of *A. arguta* relaxes after the three treatments, and the cytosol is more easily solubilized and migrated, increasing the content of TSS [20]. There was no significant difference in TSS between *A. arguta* of AT and RWT (*p* > 0.05). TAC was highest in *A. arguta* of RWT, and there was no significant difference between AT, UT, and HPPT (*p* > 0.05), and MT was the lowest. These increases are attributed to the rapid cell wall/membrane disruption caused by microwave dielectric heating, ultrasonic cavitation, and high-pressure-induced structural changes, which promote the release of intracellular soluble solids. By contrast, the slower thawing processes of AT and RWT did not significantly alter TSS levels. The reduced TAC in MT may be related to accelerated acid degradation under localized high temperatures, while the relatively stable TAC in RWT could be explained by its milder thawing conditions that minimized acid leaching and degradation.

#### 3.2.2. Drip Losses

When thawing, the ice crystals inside the food will be excessively watery, and part of the water will be absorbed by the tissue cells, while the unabsorbed will seep out, resulting in the loss of juices [37]. Previous studies on small berries have shown that drip loss, caused by cell structure damage during thawing, directly affects fruit texture and color—severe drip loss leads to loose texture and browning, while mild drip loss helps maintain good texture and color stability [38]. Meanwhile, enhanced antioxidant activity, as reported in prior research, can protect cell membranes from oxidative damage, thereby reducing drip loss and further preserving the texture and color of fruits [39]. Some water-soluble nutrients will flow out with the unabsorbed water, which will not only cause the fruit quality to be reduced, but will also provide nutrients to microorganisms, accelerating fruit rotting speed [40]. Therefore, drip loss is widely regarded as a key indicator for evaluating the quality and nutritional retention of frozen foods after thawing. Table 1 shows that the drip loss of *A. arguta* in the UT group was significantly lower (*p* < 0.05) than that of the other four thawing methods. This was due to the fact that since the cavitation effect during ultrasonic defrosting relies mainly on the mechanical vibration of the sound waves rather than direct heating, the temperature of the whole process is milder. This meant that the surface of the fruit was not overheated and was able to uniformly break down the ice crystals without harming the tissue cells of *A. arguta*, thus reducing drip losses. In addition, the energy generated by the rupture and collapse of the tiny bubbles produced during acoustic cavitation is not only effective in promoting uniform water distribution, but also in preventing widespread cell wall or membrane disruption [41]. The drip loss during microwave thawing was also lower. However, it has been found that the superheating phenomenon that occurs when microwave power is too high can cause severe drip losses [20]. The most severe loss of dripping water was caused by *A. arguta* at HPPT, which may be due to excessive pressure resulting in the destruction of tissue cells unable to absorb water. A similar phenomenon was observed during high-pressure thawing of cantaloupes [42]. The AT and RWT groups exhibited higher drip loss in *A. arguta* compared to the other three treatments, an outcome potentially attributable to their prolonged thawing duration. Overall, higher drip loss implies poorer texture and loss of nutrients in *A. arguta*. Therefore, UT is a promising method to reduce drip loss in *A. arguta*.

#### 3.2.3. Water Distribution

**Figure 2:**
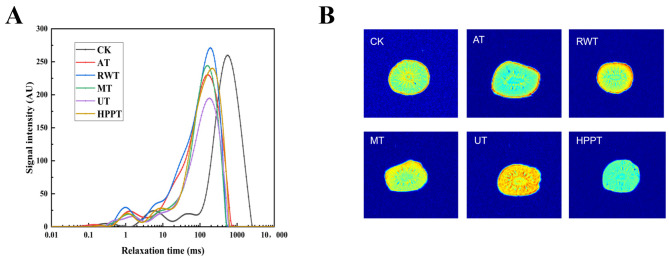
Water distribution in frozen *A. arguta* under different thawing conditions (*n* = 3, with 6 fruits per replicate). (**A**): T2 inversion spectrum. (**B**): pseudo-color map. different letters (Abbreviations: CK: unfrozen *A. arguta*; AT, air thawing; RWT, running water thawing; MT, microwave thawing; UT, ultrasonic thawing; HPPT, high-pressure processing thawing).

Low-field nuclear magnetic resonance (LF-NMR) technology is widely used to measure water molecule mobility in food tissues due to its rapid and non-destructive advantages [43]. T2 relaxation time is closely related to the water-holding properties of food tissues, and it can intuitively respond to the mobility of water [44]. The T2 relaxation inversion spectra of water in frozen *A. arguta* by different thawing methods are shown in Figure 2A. Three types of relaxation peaks existed in *A. arguta*; from left to right, they were bound water T21 (0.01–0.3 ms), weakly bound water T22 (0.3–3 ms), and free water T23 (3–1000 ms). It is obvious from the signal intensity of the relaxation peaks that the signal intensity of T23 is higher, which indicates that *A. arguta* contains more free water after thawing. This difference is mainly due to the fact that the ice crystals produced during the freezing-thawing process break the hydrogen bonds between the tissue molecules, thus increasing the mobility of the water in *A. arguta* and decreasing the ability of the water to bond with the intermolecular bonds [45]. The T23 values of RWT *A. arguta* were significantly higher than the other four thawing methods (*p* < 0.05), which indicated that RWT *A. arguta* was more likely to lose free water. In contrast, the signal intensity of the T23 relaxation peak of UT was weaker, indicating that UT could better retain bound water in *A. arguta*.

Pseudo-color maps obtained from MRI analysis of *A. arguta* after treatment with different thawing methods are shown in Figure 2B. The contrast seen in proton density-weighted images arises from the inherent variations in proton density among different tissues. In a pseudo-color image, regions exhibiting warmer colors (such as yellow or red) typically indicate a higher concentration of hydrogen protons, which correlates with increased water content in the underlying tissue [46]. As shown in the figure, HPPT-treated *A. arguta* exhibits extensive blue areas, indicating reduced bound water content (bound water peak area A = 356.96). This phenomenon can be attributed to rapid internal water evaporation caused by external high pressure. Additionally, bound water content in the seed core of *A. arguta* significantly decreased after AT treatment (bound water peak area A = 557.44). Following RWT treatment, the epidermal water content of *A. arguta* increased, resulting from the fruit peel absorbing water and facilitating external moisture penetration into the fruit interior. The pseudo-color image after MT treatment shows uneven color distribution, with peak ratios for bound water and free water at 5.82% and 94.18%, respectively, corresponding to the non-uniformity of microwave heating. Conversely, UT-treated *A. arguta* exhibited distinct and uniformly distributed red regions, with peak ratios for bound water, weakly bound water, and free water at 2.32%, 3.64%, and 94.03%, respectively. The results indicate that UT treatment better maintained the moisture status of *A. arguta*. Finally, the effects of the five thawing methods on the moisture distribution of *A. arguta* were analyzed using T2 relaxation inversion spectra and pseudo-color images, revealing that UT demonstrated significant advantages over the other four thawing methods.

### 3.3. Nutrients

#### 3.3.1. Pigment Content

**Figure 3:**
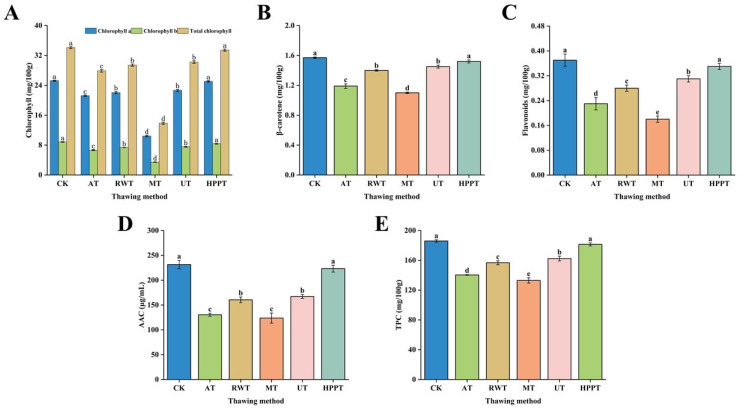
Nutritional composition of frozen *A. arguta* treated with different thawing methods (*n* = 3, with 6 fruits per replicate). (**A**): chlorophyll content. (**B**): β-carotene content. (**C**): flavonoid content. (**D**): AAC. (**E**): TPC. Error bars represent the standard deviation of triplicate measurements (*n* = 3). Values followed by different lowercase letters differ significantly (*p* < 0.05) in comparisons between samples of different concentrations. (Abbreviations: CK: unfrozen *A. arguta*; AT, air thawing; RWT, running water thawing; MT, microwave thawing; UT, ultrasonic thawing; HPPT, high-pressure processing thawing).

Chlorophylls, β-carotene, and flavonoids are essential metabolites as natural pigments that give fruits their attractive colors [47]. The pigment content of the fruit directly affects the color change in the fruit and is a fine parameter in the processing of the product. The impacts of various thawing techniques on the chlorophyll, β-carotene, and flavonoids of *A. arguta* are presented in Figure 3A–C. As the most abundant natural photosensitive pigment in *A. arguta*, chlorophyll serves as a key index reflecting color variation. Prior to thawing, no significant difference in chlorophyll content was observed between the HPPT and CK groups (*p* > 0.05). High-pressure treatment can effectively disrupt fruit cell walls, thus promoting the extraction of bioactive components, but the chlorophyll molecules inside the cells may not undergo significant changes if they are not directly exposed to the external environment or subjected to intense physical damage. There was no significant difference in chlorophyll content for *A. arguta* after RWT and UT treatments (*p* > 0.05). The amount of chlorophyll was much lower after thawing in air and microwaves. Longer thawing times or higher temperatures may have denatured the proteins that stabilize chlorophyll, resulting in chlorophyll loss [48].

β-carotene has an antioxidant effect, but more conjugated double bonds in the structure make it easy to oxidative degradation in heat treatment, acid, oxygen, light, and other conditions that are easy to isomerize [49]. The β-carotene content of *A. arguta* differed between thawing methods. The amount of β-carotene in *A. arguta* was most similar to that in the CK group after HPPT treatment. This might be because HPPT did not damage the conjugated double bond made up of isoprene residues [50]. Previous studies have found that carotenoids are susceptible to oxidation and structural changes when oxygen and high temperatures are applied [51]. This is why the β-carotene content of *A. arguta* after AT and MT treatments was significantly lower (*p* < 0.05) than that of other thawing methods. The effect of different thawing methods on the flavonoid content of *A. arguta* was consistent with the β-carotene trend, with HPPT better retaining the flavonoids of *A. arguta*, and MT treatment resulting in less flavonoids in *A. arguta*. Despite the same thermal thawing, *A. arguta* treated with UT had a higher content of flavonoids than MT. This may be due to the fact that UT can provide more suitable temperatures conducive to the extraction of flavonoids from *A. arguta*, whereas MT produces excessively high temperatures, leading to degradation of flavonoids.

#### 3.3.2. AAC and TPC

Ascorbic acid and polyphenols are both natural antioxidants found in berries and are highly effective in scavenging free radicals from the body. Ascorbic acid can participate in various biochemical reactions with coenzymes or coenzyme components and plays a crucial role in maintaining and regulating body metabolism [52]. Ascorbic acid, being a water-soluble compound, is prone to degradation under oxygen and high-temperature conditions. AAC in *A. arguta* with different thawing methods is shown in Figure 3D. The AAC of *A. arguta* treated with AT and MT was significantly lower than that of the CK group (*p* < 0.05), respectively, and statistically similar AAC values were found in *A. arguta* under RWT and UT conditions (*p* > 0.05). Compared with the CK group, the AAC of *A. arguta* under HPPT treatment exhibited no significant difference, with a value of 223.22 ± 5.66 μg/mL. The same conclusion was obtained in a study using HPP to treat pineapple juice [51]. Results presented in Figure 3E demonstrated that different thawing treatments exerted significant effects on the TPC of *A. arguta* (*p* < 0.05), in descending order of HPPT, UT, RWT, AT, and MT. HPPT-treated *A. arguta* exhibited the highest polyphenol content (181.41 ± 6.16 mg/100 g), which might result from the pressure destroying the structure of the cell wall and membrane [53]. This improved the extraction of polyphenols from *A. arguta*. The lower TPC of *A. arguta* after MT treatment (133.00 ± 4.59 mg/100 g) was due to the fact that polyphenols are heat-sensitive substances; ultrasound-induced high temperatures can cause significant damage to them.

### 3.4. Sensory Analysis

#### 3.4.1. Color

**Figure 4:**
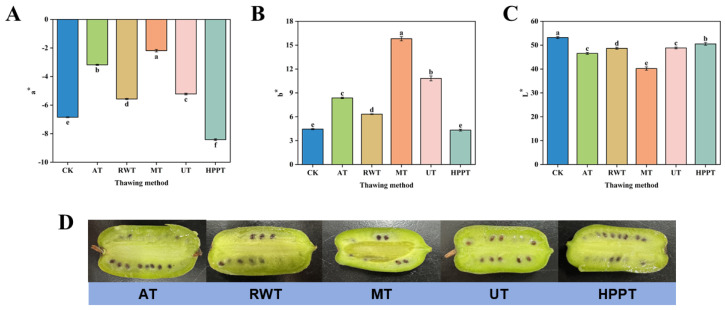
The color of frozen *A. arguta* treated with different thawing methods (*n* = 3, with 6 fruits per replicate). (**A**): a* values. (**B**): b* values. (**C**): L* values. (**D**): Visualization of thawed *A. arguta*. Error bars indicate the standard error between parallel samples (*n* = 3). Different lowercase letters indicate significant differences (*p* < 0.05) in comparisons between samples of different concentrations. (Abbreviations: CK: unfrozen *A. arguta*; AT, air thawing; RWT, running water thawing; MT, microwave thawing; UT, ultrasonic thawing; HPPT, high-pressure processing thawing).

Color represents the most critical sensory property of berries, which directly influences consumer preference, acceptance, and purchasing decisions [54]. The color of *A. arguta* was differentially affected by various thawing treatments. The variation in CIELAB parameters of *A. arguta* treated by the five thawing methods is shown in Table 2 and Figure 4. a* values represent the red-green color difference, with a positive value of a* indicating redness and a negative value indicating greenness. The a* values for the five thawing methods varied from −8.42 to −2.19, all of which were negative, indicating that the *A. arguta* had a green bias in color. The a* values of *A. arguta* in the MT group were significantly higher (*p* < 0.05) than those of the other four sample groups. The smallest a* value was for the HPPT *A. arguta*, with smaller a* values indicating a darker green coloration of the fruit, which can be attributed to the efficient release of chlorophyll from the intracellular space by the HPP treatment [55]. The b* value represents the yellow-blue difference, with a positive value of b* being the degree of yellowness and a negative value being the degree of blueness. The b* values, in descending order, were MT, UT, AT, RWT, and HPPT. All b* values were positive, suggesting that *A. arguta* tended to exhibit a yellowish hue. MT and UT *A. arguta* have large b* values and yellowish color, which is due to the fact that microwave and ultrasound are thermal defrosting, and the elevated temperatures during processing lead to the destruction of pigments such as chlorophyll. L* values are associated with fruit brightness. Ultra-high pressure treatment facilitates tissue breakdown, thereby generating finer particles with greater surface area, thereby enhancing light scattering [56]. Therefore, *A. arguta*, treated with HPPT, had higher L* values. There was no significant difference (*p* > 0.05) in L* values of *A. arguta* after RWT and UT treatments. MT-treated *A. arguta* had the smallest L*, which may be related to the increase in unstable particles in the fruit due to high temperatures. To provide a more comprehensive color evaluation, hue angle (h°) and chroma (C) were calculated from a and b. HPPT exhibited the highest h° (152.8°), even slightly higher than fresh CK (146.95°), confirming the strongest green hue. In contrast, MT gave the lowest h° (97.85°), indicating a pronounced shift from green to yellow. Regarding chroma, MT had the highest C* (15.96), approximately double that of CK (8.17), suggesting enhanced color saturation despite the loss of greenness. UT (12.02) and HPPT (9.46) showed intermediate chroma, while AT (8.95) and RWT (8.43) were similar to CK. These results demonstrate that thawing methods differentially affect not only the lightness and individual color components but also the overall hue and saturation of *A. arguta*. According to color measurement principles in food systems, color parameters including lightness (L*), red–green coordinate (a*), yellow–blue coordinate (b*), hue angle (h°), and chroma (C*) together constitute an objective and reproducible evaluation of visual quality, rather than relying on single coordinates alone [57]. Hue angle reflects the dominant visual hue, where higher values correspond to green and lower values indicate a shift toward yellow [58]. Chroma represents color saturation or intensity; higher chroma signifies more vivid and saturated coloration, which is closely associated with consumer-perceived freshness and appearance quality. Changes in hue and chroma during processing are typically related to pigment degradation, structural modification of tissue, and light-scattering properties, all of which determine the final visual appearance of fruit products [59]. In the present study, the significant variations in h° and C* among thawing treatments confirmed that different thawing strategies altered not only individual color coordinates but also the perceptual hue and saturation characteristics of *A. arguta*, which is essential for comprehensive quality assessment.

#### 3.4.2. Textural Properties

Texture is an important indicator of food flavor and quality. This study compared the influences of various thawing treatments on the texture properties of *A. arguta* by evaluating its hardness, adhesiveness, cohesiveness, springiness, and chewiness. As shown in Table 3, there was no significant difference in cohesiveness between the five thawing treatments (*p* > 0.05), indicating that thawing does not change the internal binding force that constitutes the texture of the food. Springiness is the height to which the food can be recovered between the first and second bites. UT-treated *A. arguta* had the greatest springiness, while HPPT-treated *A. arguta* had the least springiness. Chewiness is defined as the energy needed to masticate solid food until it reaches a suitable state for swallowing. It is a combination of hardness, cohesiveness, and elasticity; within a certain range, the greater its value, the better the taste of the food. In this study, UT and MT treatments resulted in hardness, springiness, and chewiness values that were statistically comparable to the fresh control (CK) (all *p* > 0.05), indicating that these two thawing methods preserved the desirable chewy texture. In contrast, HPPT treatment yielded significantly lower hardness, springiness, and chewiness than CK, UT, and MT (*p* < 0.05), making the fruit the softest and easiest to chew. No significant difference in cohesiveness was observed among any treatments (*p* > 0.05). Notably, UT treatment maintained hardness statistically similar to fresh CK (*p* > 0.05) and significantly higher than most other thawing methods (*p* < 0.05). This favorable effect can be explained by the ultrasonic cavitation effect, which gently disrupts internal ice crystals and reduces tissue damage during thawing. The hardness and stickiness of *A. arguta* after MT treatment are higher, which is due to the fact that microwave treatment increases the glucose and pectin content of *A. arguta*, and part of the glucose and pectin exudes from the peel, thus making the texture of *A. arguta* hard and the surface skin sticky [60]. Taken together, the texture of *A. arguta* was differentially influenced by various thawing methods, with UT- and MT-treated *A. arguta* exhibiting harder, more elastic, and more energy required for chewing, while HPPT-treated soft *A. arguta* exhibited the softest, weakest elasticity, and easier chewing.

### 3.5. Microstructure

**Figure 5:**
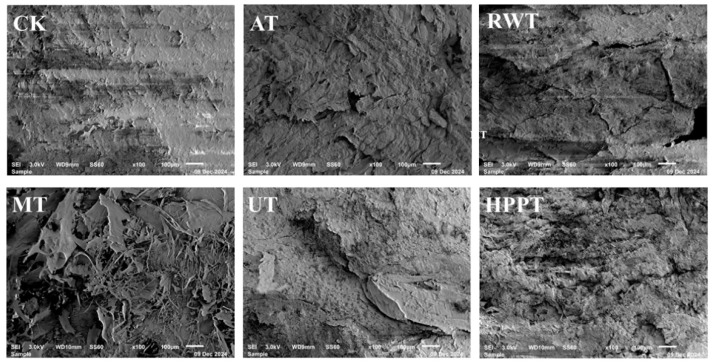
Microstructure of frozen *A. arguta* treated with different thawing methods. (magnification: 100×) (Abbreviations: CK: unfrozen *A. arguta*; AT, air thawing; RWT, running water thawing; MT, microwave thawing; UT, ultrasonic thawing; HPPT, high-pressure processing thawing).

Figure 5 shows the effects of different thawing methods on the microstructure of *A. arguta*. Moisture migration and melting of ice crystals during the thawing process directly affected the microstructure of *A. arguta*. AT, RWT, and MT samples had rougher surfaces and showed some cracks and inhomogeneous fragments. This structural disruption is highly associated with the water-holding capacity of the tissue [61]. The MT group showed marked tissue fragmentation and produced irregular filamentous structures. In contrast, the surface of the UT sample is essentially free of cracks and exhibits a more compact texture. It is interesting to look at the HPPT samples’ microstructure, which shows broken fibers wrapped around pores and uneven blocky structures. These features result in a softer texture and fewer water-holding properties in the sample. Overall, ultrasonic treatment hardly affects *A. arguta* tissues, and high-pressure treatment causes severe tissue damage. The tighter the tissue structure, the better the water retention ability and hardness of the fruit. Thus, the SEM findings were in accordance with those obtained from water status and texture analyses.

### 3.6. Antioxidant Capacity

**Figure 6:**
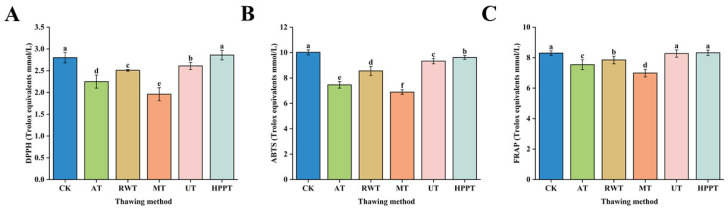
Antioxidant capacity of frozen *A. arguta* treated with different thawing methods (*n* = 3, with 6 fruits per replicate). (**A**): DPPH. (**B**): ABTS. (**C**): FRAP. Error bars indicate the standard error between parallel samples (*n* = 3). Different lowercase letters indicate significant differences (*p* < 0.05) in comparisons between samples of different concentrations. (Abbreviations: CK: unfrozen *A. arguta*; AT, air thawing; RWT, running water thawing; MT, microwave thawing; UT, ultrasonic thawing; HPPT, high-pressure processing thawing).

Due to the complexity and diversity of antioxidant components and action mechanisms, to ensure an accurate and comprehensive comparison of how various thawing treatments influence the antioxidant capacity of *A. arguta*, three methods, namely, DPPH, ABTS, and FRAP, were selected for the comprehensive evaluation of antioxidant capacity. The antioxidant capacity of small berries is mainly attributed to endogenous bioactive substances such as flavonoids, vitamin C, and SOD. During thawing, different thawing treatments cause varying degrees of cellular structural damage, resulting in the leakage and loss of intracellular antioxidant substances. Meanwhile, cell rupture enables oxidative enzymes to fully contact their substrates, accompanied by increased oxygen infiltration and excessive accumulation of free radicals, which ultimately leads to significant differences in antioxidant activity among different thawing groups. Results presented in Figure 6 revealed significant differences (*p* < 0.05) in the antioxidant capacity of *A. arguta* under different thawing methods. DPPH, ABTS, and FRAP of *A. arguta* before freezing were 2.80 ± 0.12, 10.02 ± 0.20, and 8.30 ± 0.15 trolox equivalents mmol/L, respectively. The DPPH, ABTS, and FRAP of HPPT *A. arguta* were 2.86 ± 0.11, 9.62 ± 0.15, and 8.32 ± 0.18 trolox equivalents mmol/L, respectively, which were markedly higher than the values obtained from the other four thawing methods (*p* < 0.05). This might be attributed to the better antioxidant capacity of *A. arguta* due to better retention or even an increase in antioxidant components by UHP treatment. In addition, UHP treatment may activate or enhance the activities of some antioxidant-related enzymes (e.g., peroxidase, superoxide dismutase, etc.). These enzymes can act as scavengers of free radicals and slow down the oxidative process both inside and outside the cell, thus enhancing the antioxidant capacity of *A. arguta* [62]. The antioxidant capacity of UT and RWT *A. arguta* was moderate compared to other methods. AT and MT *A. arguta* showed poorer antioxidant capacity due to prolonged exposure to air and high temperatures.

### 3.7. Correlation Analysis

**Figure 7:**
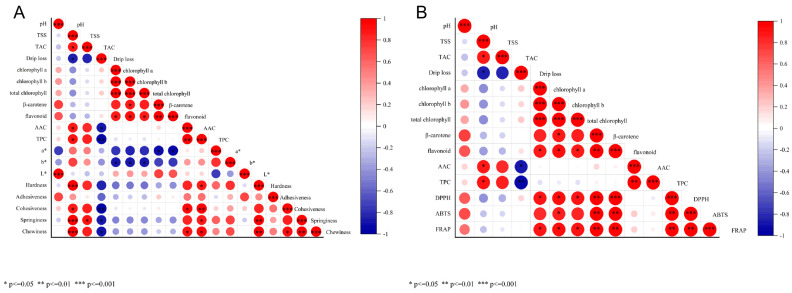
Correlation analysis of physicochemical characteristics and nutrient composition of *A. arguta* with different thawing methods on sensory quality (**A**) and antioxidant capacity (**B**) (*n* = 3, with 6 fruits per replicate).

In order to investigate the effects of physicochemical characteristics (pH, TSS, TAC, drip loss) and nutrient composition (chlorophyll a, chlorophyll b, total chlorophyll, β-carotene, flavonoids, AAC, TPC) of *A. arguta* on sensory quality (a*, b*, L*, hardness, adhesiveness, cohesiveness, springiness, chewiness) and antioxidant capacity (DPPH, ABTS, FRAP), correlation analyses are necessary (Figure 7). In the figure, blue represents negative correlations while red represents positive ones. The strength of the correlations was classified as follows: very weak (0–0.19), weak (0.2–0.39), moderate (0.4–0.59), strong (0.6–0.79), and very strong (0.8–1.0) [63]. TSS showed significant positive correlations with hardness, elasticity, and chewiness (*p* < 0.05, R^2^ = 0.9918–0.9944); it also exhibited strong positive correlations with TAC and cohesiveness (*p* < 0.05, R^2^ = 0.8504–0.8823). This is primarily because increased TSS typically elevates fruit sugar content, which enhances fruit hardness and produces a denser flesh structure, thereby improving chewiness and elasticity. Nutrients show a strong positive correlation with each other. This suggests that there are certain associations between the various nutrients, which influence the variations in their contents. The negative correlation between nutrient composition and color confirms that *A. arguta* nutrients, especially pigmented components, have a forceful influence on its color. Among them, β-carotene and flavonoids significantly affected the a* values (*p* < 0.05, R^2^ = 0.8718–0.8791), and chlorophyll significantly affected the b* values of *A. arguta* (*p* < 0.05, R^2^ = 0.8479–0.8508). As shown in Figure 7B, a highly significant correlation (*p* < 0.01) was exhibited between antioxidant capacity. Interestingly, drip loss showed significant correlation (*p* < 0.05, R^2^ = 0.8504–0.8823) for DPPH and FRAP. This may be due to the fact that most of the biochemicals with antioxidant capacity are water-soluble, and water penetration on the fruit surface results in a significant loss of these bioactive components, leading to changes in antioxidant capacity. Chlorophyll, β-carotene, and flavonoids showed significant correlations with DPPH, ABTS, and FRAP (*p* < 0.05, R^2^ = 0.8545–0.8989). Chlorophyll, β-carotene, and flavonoids frequently act synergistically as natural antioxidants in plants. Their antioxidant activity is closely linked to the conjugated double bonds and phenolic hydroxyl structures within their molecules. They can react with radicals and oxidants in antioxidant assays such as DPPH, ABTS, and FRAP through various mechanisms (e.g., electron donation, radical scavenging), thereby exhibiting significant correlations. It is noteworthy that AAC, a representative antioxidant active component in fruits, showed a relatively weak correlation with the DPPH and ABTS radical scavenging abilities of *A. arguta* (*p* > 0.05, R^2^ = 0.1464–0.2998). Ascorbic acid (AA), a water-soluble vitamin, possesses strong antioxidant properties but exhibits poor stability in external environments. Particularly during freezing and thawing processes, AAC undergoes significant changes. The potency of DPPH and ABTS radical scavenging depends on an antioxidant’s electron-donating capacity. AA exerts its antioxidant effect by providing electrons to neutralize radicals. However, AA degradation after thawing reduces its electron-donating capacity, thereby diminishing its contribution to radical scavenging.

## 4. Conclusions

In this study, the effects of five thawing methods (AT, RWT, MT, UT, and HPPT) on the quality of frozen *A. arguta* were investigated. The results showed that RWT, MT, UT, and HPPT significantly reduced the thawing time compared with the conventional air thawing method. MT severely damaged the nutrients and color of the fruits. HPPT was the most efficient thawing method, which retained the nutrients better, but the water retention of the fruits was reduced, and the texture became softer. Texture and scanning electron microscopy analyses showed that UT-treated *A. arguta* had a firmer tissue structure, which was more conducive to water retention and affected consumer palatability. Additionally, *A. arguta* treated with US and HPPT exhibited enhanced antioxidant capacity. In summary, if the goal is to maximize nutrient retention, HPPT is undoubtedly a more ideal choice; however, in scenarios requiring large-scale processing or extended shelf life, UT offers undeniable advantages. Based on the results of this study, future research should focus on deepening the molecular mechanisms by which different thawing methods regulate antioxidant capacity, optimizing the core processing parameters of UT and HPPT, developing UT-HPPT combined thawing technology and an integrated quality control system for the freeze–thaw-storage whole chain, and improving the quality evaluation system and industrial adaptation research. In terms of guiding principles, it is necessary to focus on the coordinated optimization of quality, balance theoretical depth and industrial practicality, emphasize interdisciplinary integration, promote the transformation of research results oriented by industrial needs, and provide support for the industrial development of frozen preservation of small berries.

## Figures and Tables

**Table 1:** Physicochemical properties of frozen *A. arguta* treated with different thawing methods.

Sample	CK	AT	RWT	MT	UT	HPPT
pH	3.6 ± 0.10 ^a^	3.5 ± 0.15 ^a^	3.7 ± 0.12 ^a^	3.6 ± 0.17 ^a^	3.6 ± 0.15 ^a^	3.7 ± 0.15 ^a^
TSS (%)	15.48 ± 0.15 ^c^	15.21 ± 0.11 ^c^	15.53 ± 0.28 ^c^	16.02 ± 0.11 ^b^	16.52 ± 0.19 ^a^	16.37 ± 0.18 ^b^
TAC (g/L)	12.15 ± 0.26 ^a^	11.17 ± 0.17 ^b^	12.26 ± 0.34 ^a^	8.61 ± 0.27 ^c^	10.62 ± 0.29 ^b^	10.87 ± 0.67 ^b^
Drip loss (%)	—	45.57 ± 0.91 ^b^	29.48 ± 0.77 ^c^	20.59 ± 0.73 ^d^	16.75 ± 1.74 ^e^	53.40 ± 2.12 ^a^

Data are expressed as mean ± standard deviation (*n* = 3, with 6 fruits per replicate). Different letters in the same row indicate significant differences between means (*p* < 0.05). (Abbreviations: CK: unfrozen *A. arguta*; AT, air thawing; RWT, running water thawing; MT, microwave thawing; UT, ultrasonic thawing; HPPT, high-pressure processing thawing).

**Table 2:** Study on the color of frozen *Actinidia arguta* treated by different thawing methods. Different lowercase letters indicate significant differences (*p* < 0.05) among different samples.

	CK	AT	RWT	MT	UT	HPPT
h°	146.95 ± 0.21 ^b^	110.8 ± 0.28 ^e^	131.35 ± 0.35 ^c^	97.85 ± 0.07 ^f^	115.75 ± 0.21 ^d^	152.8 ± 0.85 ^a^
C*	8.17 ± 0.02 ^f^	8.95 ± 0.01 ^d^	8.43 ± 0.02 ^e^	15.96 ± 0.05 ^a^	12.02 ± 0.06 ^b^	9.46 ± 0.03 ^c^

**Table 3:** Textural characterization of frozen *A. arguta* treated with different thawing methods.

Sample	CK	AT	RWT	MT	UT	HPPT
Hardness/N	26.32 ± 1.35 ^a^	19.14 ± 1.79 ^bc^	21.38 ± 4.05 ^ab^	25.88 ± 0.40 ^a^	27.48 ± 1.75 ^a^	14.18 ± 2.31 ^c^
Adhesiveness/mJ	0.15 ± 0.03 ^ab^	0.06 ± 0.06 ^b^	0.15 ± 0.04 ^ab^	0.16 ± 0.01 ^a^	0.15 ± 0.02 ^ab^	0.13 ± 0.01 ^ab^
Cohesiveness	0.67 ± 0.05 ^a^	0.54 ± 0.11 ^a^	0.61 ± 0.09 ^a^	0.58 ± 0.10 ^a^	0.64 ± 0.03 ^a^	0.52 ± 0.03 ^a^
Springiness/mm	3.68 ± 0.09 ^a^	3.43 ± 0.04 ^ab^	3.48 ± 0.09 ^ab^	3.55 ± 0.10 ^a^	3.61 ± 0.02 ^a^	3.27 ± 0.14 ^b^
Chewiness/mJ	65.21 ± 5.36 ^a^	35.08 ± 9.85 ^bc^	46.03 ± 6.84 ^abc^	53.29 ± 11.05 ^ab^	63.30 ± 1.82 ^a^	23.89 ± 1.34 ^c^

Data is expressed as mean ± SD (*n* = 3, with 6 fruits per replicate). Values within the same row followed by different letters are significantly different (*p* < 0.05). (Abbreviations: CK: unfrozen *A. arguta*; AT, air thawing; RWT, running water thawing; MT, microwave thawing; UT, ultrasonic thawing; HPPT, high-pressure processing thawing).

## Data Availability

The original contributions presented in this study are included in the article. Further inquiries can be directed to the corresponding author.
